# Designing effective visualizations of habits data to aid clinical decision making

**DOI:** 10.1186/s12911-014-0102-x

**Published:** 2014-11-30

**Authors:** Joost de Folter, Hulya Gokalp, Joanna Fursse, Urvashi Sharma, Malcolm Clarke

**Affiliations:** Brunel University, Uxbridge, UB8 3PH UK; Chorleywood Health Centre, 15 Lower Rd, Chorleywood, Rickmansworth, WD3 5EA UK

**Keywords:** Visualization, Decision making, User centered design, Habits data, Feature extraction

## Abstract

**Background:**

Changes in daily habits can provide important information regarding the overall health status of an individual. This research aimed to determine how meaningful information may be extracted from limited sensor data and transformed to provide clear visualization for the clinicians who must use and interact with the data and make judgments on the condition of patients. We ascertained that a number of insightful features related to habits and physical condition could be determined from usage and motion sensor data.

**Methods:**

Our approach to the design of the visualization follows User Centered Design, specifically, defining requirements, designing corresponding visualizations and finally evaluating results. This cycle was iterated three times.

**Results:**

The User Centered Design method was successfully employed to converge to a design that met the main objective of this study. The resulting visualizations of relevant features that were extracted from the sensor data were considered highly effective and intuitive to the clinicians and were considered suitable for monitoring the behavior patterns of patients.

**Conclusions:**

We observed important differences in the approach and attitude of the researchers and clinicians. Whereas the researchers would prefer to have as many features and information as possible in each visualization, the clinicians would prefer clarity and simplicity, often each visualization having only a single feature, with several visualizations per page. In addition, concepts considered intuitive to the researchers were not always to the clinicians.

**Electronic supplementary material:**

The online version of this article (doi:10.1186/s12911-014-0102-x) contains supplementary material, which is available to authorized users.

## Background

Over the last 25 years the percentage of the population aged 65 and over has increased. By 2034 the number of people aged 85 and over is projected to account for 5% of the total population. Along with this increase comes increased clinical need. Many amongst this ageing population are living with one or more long term chronic conditions [1].

The EU funded project inCASA (Integrated Network for Completely Assisted Senior citizen’s Autonomy) [2] developed an integrated service delivery model that combined health and social care services to provide a coordinated response to the needs of frail elderly people with long term conditions. The integrated service was driven by the needs of health and social care and information about the patient and data from remote monitoring was shared and exchanged between the primary care team and social services.

One way of promoting independent living is by monitoring daily habits of the elderly. Changes in daily habits can provide important information regarding well-being, functional capability, cognitive ability, loss of autonomy/independence, deterioration in health status, or progress of an existing illness [3]. Although there are a significant number of studies and reviews on the monitoring of chronic diseases, with themes of technology or treatment effectiveness [4-9], there are only a few reviews on monitoring of daily activities in elderly patients [10,11] and, as found in [12], there are no studies that sought to correlate activity, physiological monitoring and clinical events.

Patients enrolled into the inCasa service were provided with integrated technology to collect both health and habits monitoring data. The devices automatically transmitted the information via a home hub and data was analyzed in order to profile user behavior and to compare variations in the activity data with the variations in the physiological parameters. Clinicians carried out initial assessment of the patient data and if necessary would refer the patient for clinical intervention, social services or other community services as required. In this paper we use the term clinician to refer to general practitioners and practice nurses. The research supports independent living and responds to the combined clinical and social needs of an elderly population.

Patients were identified using a combination of health and social care registers. The level of frailty was assessed using the Edmonton Frail Scale [13] in addition to a number of other clinical measures. A total of 44 people were recruited into the service and 36 completed a minimum of 30 days of monitoring. 61% of those enrolled into the service were determined as being of average frailty and 27% as being very frail, leaving the remaining 12% as not frail.

Extensive research has been undertaken on sleep and activity patterns and their significance [14-16]. However it is desirable to obtain sleep and activity data in a way that is unobtrusive and does not rely on the active participation of the subjects, especially if this involves patients with a health problem. Although some research has been done on automatic collection of data, most systems have been established in purpose facilities rather than deployed with patients living in their own home, and not on a significant scale [12]. Clearly there are challenges. One of these is developing a platform that can be deployed at low cost, having devices for both activity and physiological monitoring, and fits well with UK health services. A cost-effective and minimally invasive solution in a real home would imply that the platform has a limited number of sensors, giving limited but adequate sensor information. The key area of research is how to determine the minimum monitoring requirements and how meaningful information may be extracted from that limited sensor data. This paper reports on the results of the development of our system, determination of data requirements, and analysis. A further important aspect of the research is how the data is transformed to enable clear and intuitive visualization for the clinicians that must use and interact with the data and make judgments on the condition of patients. This is also an area where little or no research has been undertaken.

In general, visualization has been widely used to enhance understanding and to promote insight into data and processes [17]. Effective visualization techniques are essential to allow patterns to be observed; that is, finding effective visualization to support the human ability to recognize complex patterns not easily detected otherwise [18].

The aim of this research was to provide the clinicians with tools to enable them to observe sensor data and determine anomalies. Our approach was to create visualizations that clinicians would consider intuitive and effective, allow them to recognize issues with a patient, and that would indicate the need for intervention and facilitate effective and expeditious decision making.

A well-established process of design taking into account subjective user perception is the User Centered Design (UCD) technique [19]. UCD allows users to be part of the process within the intended context and facilitate the design to evolve. In this study the participants are the clinicians, and what is taken into account for improving the design is their perception of what they consider effective visualizations.

The visualizations in this study are based on medical sensor data. This includes processing the sensor data to extract and present significant features. The sensors that were deployed in the home of each patient were at most two motion sensors, or one motion and one bed usage sensor.

## Methods

### Data acquisition

Data was acquired from frail and elderly patients living in their own home and in otherwise normal circumstances and included both data for habits monitoring and physiological data to monitor their chronic disease(s). The system contained a combination of sensor types (*Agents)* for the habits and physiological monitoring, and each connected wirelessly to a single hub (*Manager*) in the home, which then transmitted data using cellular communication generally known as General Packet Radio Service (GPRS) to a central database. All communication from sensors to hub was based on the IEEE 11073 personal health device (PHD) standards [20] and ZigBee Health Care Profile (ZHCP) [21]. Communication from the hub was based on the Integrated Health Enterprise Patient Care Device (IHE-PCD01), a profile of Health Language 7 (HL7) [22].

**Figure 1:**
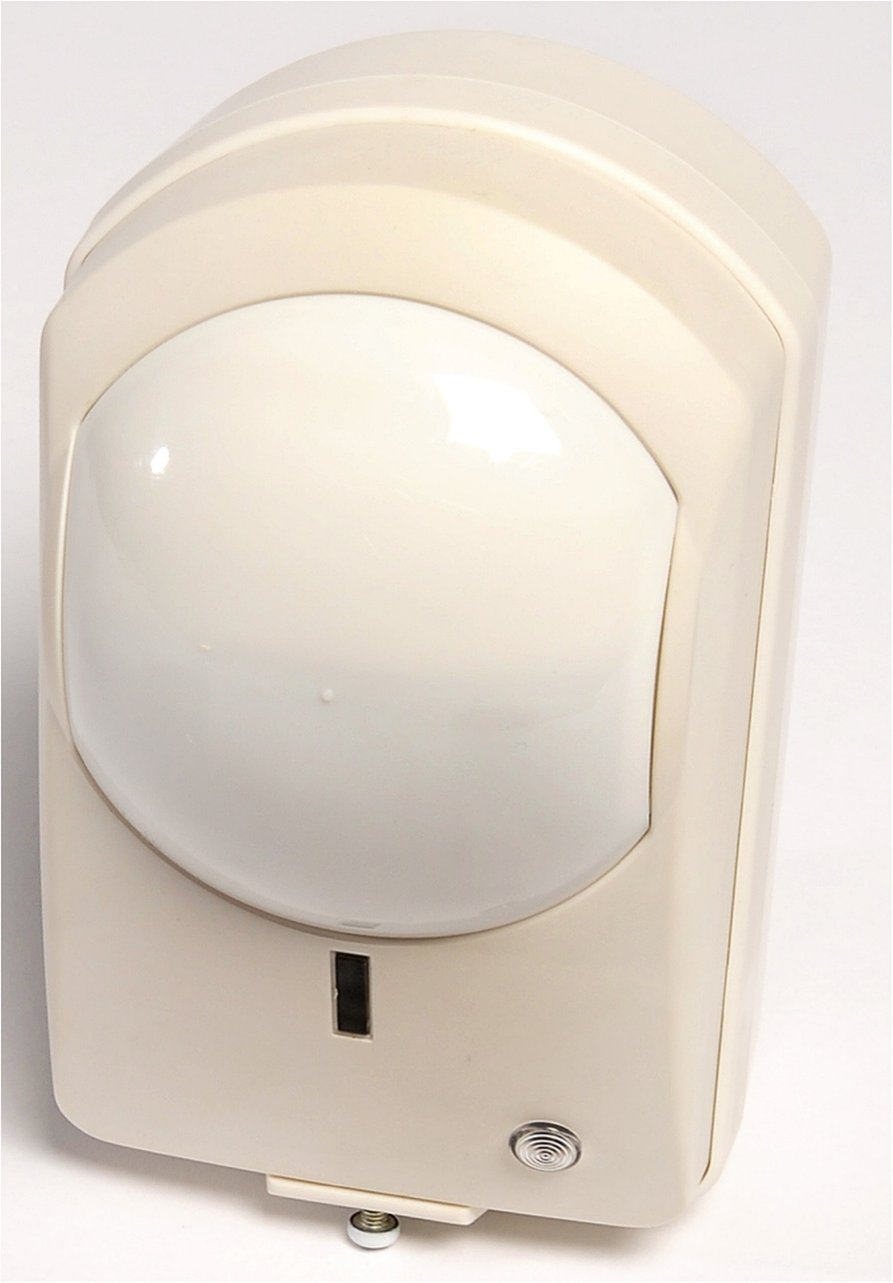
**Photo of motion sensor.**

Two types of sensor were used for habits monitoring. One or more motion sensors recorded movement within the field of vision. The sensor used in this study (Figure 1) had a dead time of 2 minutes, during which time further movement was ignored. This would reduce both the number of repetitions of movement, and thus the amount of data that had to be transmitted and battery power. The location for this sensor was determined to be the place where most activity was observed during the day, typically the living room. We determined that the bed should not be in the field of vision of the sensor, so any motion in bed would be excluded.

**Figure 2:**
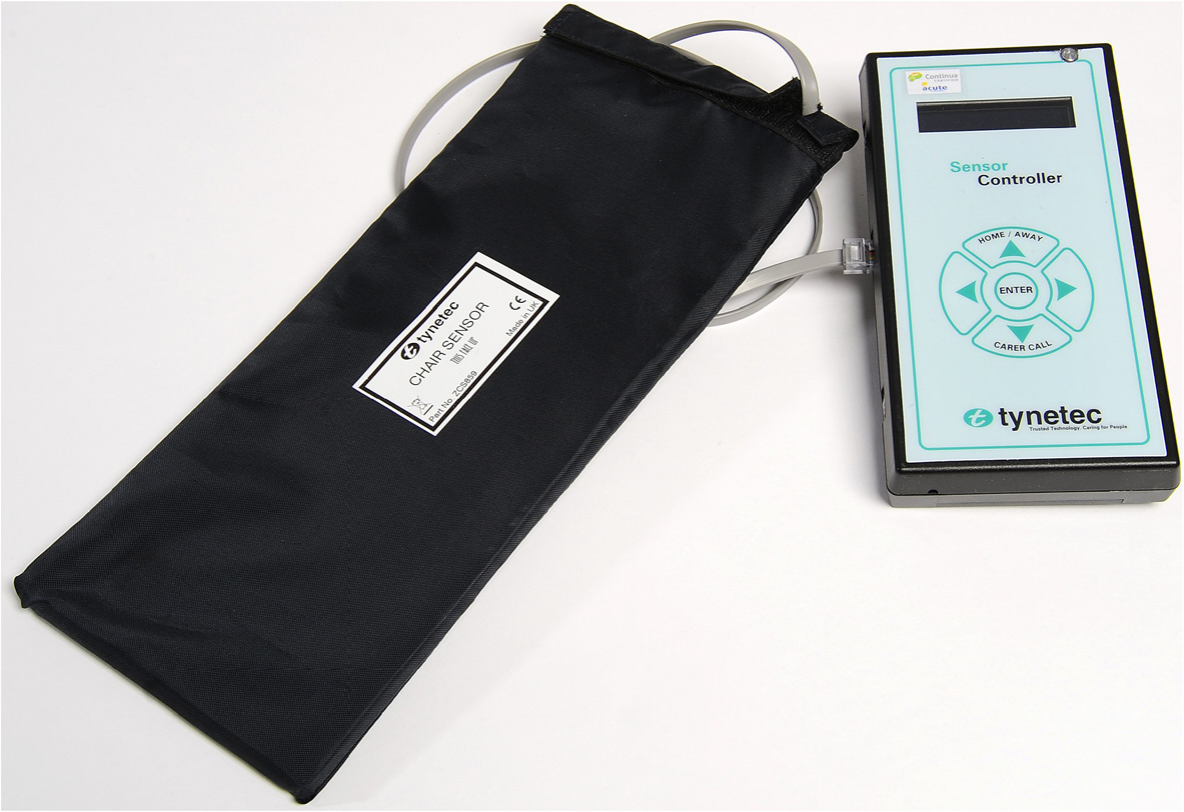
**Photo of bed usage sensor: pressure pad (left) and control unit (right).**

Bed usage sensors were implemented as a calibrated pressure sensor beneath the mattress, which would sense presence or absence. Occasionally the sensor was placed in a chair instead. The sensor (Figure 2) could be configured with the minimum time for which a change on the sensor must be registered before a message was sent to indicate ‘usage start’ and ‘usage end’. We found 30 seconds to be satisfactory. The usage sensor was of commercial design and modified to take the IEEE 11073 radio module to allow integration to the platform.

**Figure 3:**
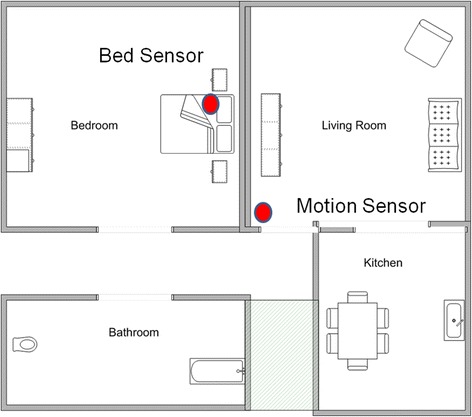
**Example of floor plan with sensor locations.**

Typical sensor locations in a home are shown in Figure 3.

### Data processing

#### Motion sensor

The motion sensor reports discrete events of motion detected in its field of view using Passive Infrared (PIR) technology. The actual sensor had a dead time of two minutes interposed between events. Although discrete sensor events can be displayed (e.g., as a series of impulses), we determined that the clinicians preferred to have a visualization that was based on a continuous smoothed function [23], where the frequency of impulses was replaced by an amplitude to represent level of activity. For this purpose we chose to represent the event data as a sampled waveform (a series of unit weighted impulses) and performed convolution with a normal Gaussian function with sigma (σ) of 15 minutes to create an activity function that provides a representation of level of activity. In practice we truncated the Gaussian function at 3σ (99% of power). The resulting time continuous function can be displayed either as a two dimensional (x-y) graph or as a one dimensional color or intensity gradient plot (x-z). This same approach can be applied to data from any usage sensor having discrete events.

### Events count

An approach based on accumulated events within a specified period was found to convey information in a quickly assimilated form. Previous studies have reported that the counts per day of events from the motion sensor reflect changes in activity level of the subject, and changes in event counts for the night time period can be associated with general well-being of the subjects [24-27]. Bed usage sensor event counts have been found to be related to the frequency of bathroom visits [25,26]. Any number of periods over which the number of motion and bed usage sensor events is counted can be chosen, for example events can be counted for each hour, each day, etc. From our work we determined that presenting the data in the three periods over a 24 hour period that correspond to specific types of activity provided clearest presentation of salient information; this being daytime activity (10:00 – 22:00), nighttime (22:00 – 06:00) and early morning and waking (06:00 – 10:00). These time periods were determined from observing patient data over a period of 6 months, to determine the periods that best represent late night activity (i.e. going to sleep) and early morning activity (i.e. waking up).

### Feature extraction

We determined that important information relating to habits and physical condition could also be determined from the raw data of the motion and usage sensors. This includes the start and end time of an activity, such as going to bed for the night or getting up, or the duration of the activity such as the total time in bed for the night. Table 1 enumerates and describes the features that we identified for the usage sensor in our work. Table 2 enumerates and describes the features identified for the motion sensor and relate to nocturnal inactivity.

**Table 1:** **Features for usage sensor**

**Feature**	**Description**
Time in bed	The (first) time in bed (after 18:00)
Time out of bed	The (last) time out of bed (before 12:00)
Mean time	The mean time of being in bed (over accumulated period in bed)
Total period in bed	Total period between time in bed and time out of bed
Accumulated period in bed (or Occupancy)	Accumulation of all periods bed was occupied
Accumulated period out of bed	Accumulated periods bed was unoccupied between time in bed and time out of bed
Continuity	Accumulated period in bed divided by Total period in bed

**Table 2:** **Features for motion sensor**

**Feature**	**Description**
Start of inactivity	The time activity ceased in the evening / night
End of inactivity	The time of first activity in the morning
Mean time of inactivity	The mean time between Start time and End time
Duration of inactivity	Total period of nocturnal inactivity

### Visualization design

**Figure 4:**
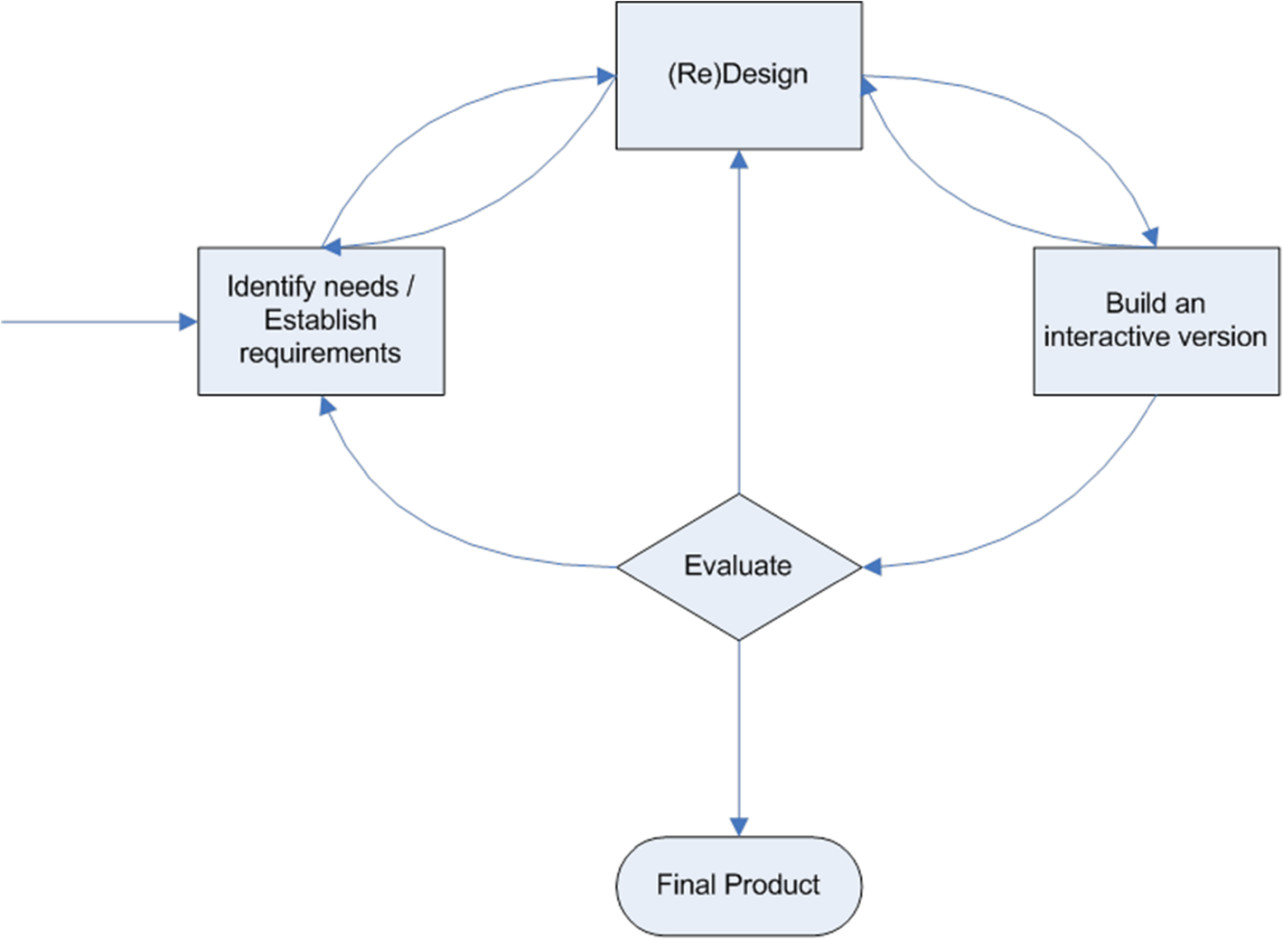
**Simple interaction (user centered) design lifecycle model [** 19 **].**

Our approach to the design of the visualization follows User Centered Design (UCD) [19] (Figure 4). In this approach, a prototype is designed and built based on initial needs or requirements. The prototype is subjected to evaluation by the users, and feedback is taken and used to refine the requirements. The cycle is repeated, often and frequently. Ideally the prototype will include aspects of interactive design so that alternatives can be quickly explored during evaluation. In our research, in place of building an interactive version we chose to provide a number of alternative static visualizations for comparison.

In our work, we held a series of informal discussions with individual clinicians to elicit initial requirements. Evaluation was performed in a succession of formal group meetings. Design proceeded by taking a number of raw data sets and forming features and creating alternative visualizations to present to the clinicians for feedback. After a number of early cycles of evaluation using static visualizations we proceeded to build an interactive version for final evaluation and to provide the basis to build the final product.

### Evaluation methods

Evaluation was undertaken using mixed methods, qualitative and quantitative. The participants of this study comprised one male doctor, one female nurse, one female research manager, and one independent female researcher. All were independent from the team carrying out the research. All the participants are senior members of a General Practitioner surgery located in an affluent area of Hertfordshire that has 6000 registered patients. Each participant has a professional experience of over 15 years in their field of expertise. All the participants have prior experience of working with remote patient monitoring technology, and consider that monitoring patients in their own homes for a physiological condition has inherent advantages.

Evaluation of the static visualizations took the form of showing each of the visualizations to the participants, collecting qualitative feedback using unstructured group discussion, and making notes of the comments. At the same time, individuals were asked to complete a score sheet that had a Likert scale to provide quantitative feedback on the perceived effectiveness of each of the visualizations, using a value between 1 (very low) and 5 (very high). An example scoring sheet is included in Additional file 1. The qualitative feedback was used as the main criteria in the UCD cycle, though the quantitative feedback formed a useful tool to assess the level of effectiveness of the visualizations.

Following the process of iterative design, the feedback was analyzed to elicit new requirements for successive redesign and re-evaluation. In general we made a single modification for consideration in each of the iterations and we show the evolution of visualizations for each respective cycle in the results. This meant that visualizations receiving feedback for improvement were taken forward to the next cycle, and those that were not considered effective and did not receive feedback for improvement were not developed further.

Ethical approval for this project was provided as part of the ethical approval sought and approved for the project inCASA from the UK National Research Ethics Committee (NREC). Written informed consent to participate in this study was gained from each participant.

## Results

### Cycle 1

Initial requirements were collected in August 2013. The main requirements were to have separate views of a summarization of activity over a period of several days, and a detailed view of any 24 hour period. In addition, each of the visualizations should be appropriate for the type of sensor data being shown.

**Figure 5:**
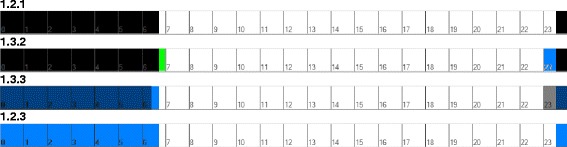
**The 24 hour visualization of usage sensor showing activity: 1.2.1: Sensor in use as black, sensor not in use as white; 1.3.2: Difference to normal pattern added shown in green and blue; 1.3.3: Actual data with normal pattern superimposed as darker tint; 1.2.3: Sensor in use as blue, sensor not in use as white.**

**Figure 6:**
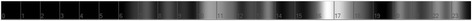
**Filtered motion sensor data (2.2.1).**

**Figure 7:**
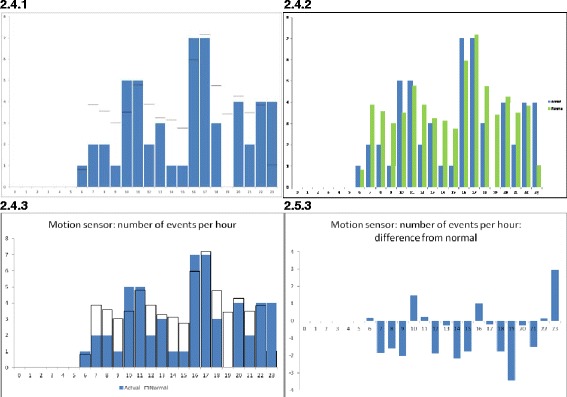
**The 24 hour visualization of motion sensor showing number of events per hour: 2.4.1: Actual data in blue, normal pattern as black lines; 2.4.2: Normal pattern added as separate green bar; 2.4.3: Actual data in blue, normal pattern as black line; 2.5.3: Showing difference between current data and normal pattern.**

In our first efforts, the visualization of events over a 24 hour period were presented as a two dimensional graph and is very similar to the raw sensor data in form. Several variants of visualization were proposed. The complete enumeration of visualizations conceived throughout this study is shown in Table 3, which furthermore defines the identifiers assigned to the visualizations. The double digit identifier is subsequently extended by a third digit, which identifies the cycle number, resulting in the format: ‘(group).(variation).(cycle)’. For the usage sensor, one of the variations simply shows *in use* (black) or *not in use* (white) (Figure 5: 1.2.1). For the motion sensor, we initially showed each motion event as a pulse on the graph (2.1.1; not shown here), but quickly adopted the continuous waveform in its place (2.3.1; not shown here), and this could be visualized as a two dimensional graph or a one dimensional color or intensity gradient plot (as shown in Figure 6: 2.2.1). An alternative form for the motion sensor (effectively a crude approximation to the continuous function) was to visualize the number of events that occurred every hour and display this as a bar graph (Figure 7: 2.4.1).

**Table 3:** **Enumeration of conceived visualizations; identifiers and corresponding descriptions**

**Identifier**	**Description**
1.1	24 hour usage line graph
1.2	24 hour usage activity simple
1.3	24 hour usage activity colors
1.4	24 hour usage activity bars
2.1	24 hour motion activity line graph
2.2	24 hour motion activity window
2.3	24 hour motion activity window graph
2.4	24 hour motion events / hour
2.5	24 hour motion events / hour differences
3.1	Days usage occupancy times
3.2	Days usage occupancy hours
3.3	Days usage occupancy only hours
3.4	Days usage non-occupancy hours
3.5	Days usage occupancy hours stacked
3.6	Days usage occupancy hours stacked areas
4.1	Days usage # events
4.2	Days usage # events / day parts
5.1	Raw usage occupancy
6.1	Days motion nocturnal inactivity times
6.2	Days motion nocturnal inactivity hours
7.1	Days motion # events
7.2	Days motion # events / day parts
8.1	Raw motion # events / hour
8.2	Raw motion activity 15 minute window
8.3	Raw motion activity 30 minute window

**Figure 8:**
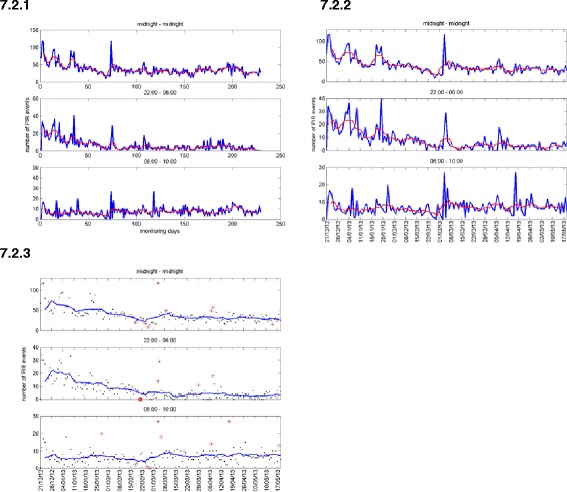
**Days visualization of motion sensor showing number of events per part of day 7.2.1: Actual data in blue and running average in (smoothed) red; 7.2.2: Showing dates on horizontal axis; 7.2.3: Showing actual data as points and running average as solid line.**

**Figure 9:**
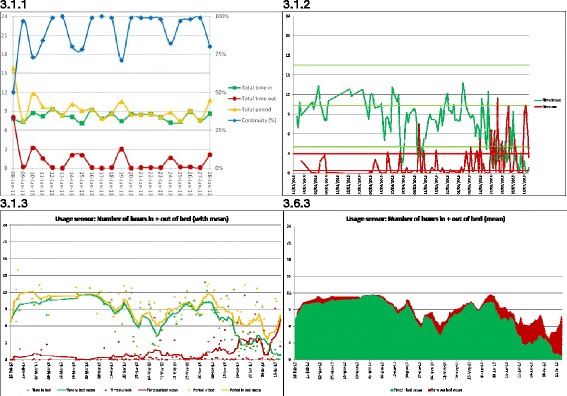
**Visualization of usage sensor showing number of hours for each day 3.1.1: Number of hours features and continuity, where Total period (yellow line) is the sum of data for in (green line) and out (red line) of bed during the expected bed period; 3.1.2: Showing only time in (green line) and out of (red line) bed during expected bed period with normal pattern and deviation threshold lines; 3.1.3: Showing running average as solid line and actual data as points for total time (yellow line), time in (green line) and time out (red line); 3.6.3: Showing simplified stacked area graph of running average values.**

To visualize the sensor data over a number of days, the data can be shown in a more summarized form, with the number of events of the motion or usage sensor accumulated for the entire day, or parts of a day, as described in section 2.2. This could then be visualized as a line graph (Figure 8: 7.2.1). Features such as “start of activity” and “duration of activity” could be found, as detailed in section 2.2, and visualized as line graphs (Figure 9: 3.1.1).

We also derived a measure of usual behavior for each patient and this was superimposed on the visualization in order to accentuate departure from usual. This usual pattern was obtained by determining the average of the daily activity of the patient over a period of 21 days. For example, the black lines in Figure 7: 2.4.1 show the usual behavior superimposed on actual activity shown in blue.

For the evaluation of the first visualizations, we found qualitative feedback using individual interviews to be the most informative. In this initial review, quantitative feedback was not collected. For the usage sensor, the participants’ feedback was that they preferred the one dimensional color graph (Figure 5: 1.2.1), and suggested that the usual pattern could be superimposed using different colors. For the motion sensor, the participants preferred a bar graph showing the hourly number of events (Figure 7: 2.4.1) as it could be compared easily with the usual pattern. There were further suggestions on how to improve the way in which the usual pattern was presented. The participants stressed that they were often looking for differences between values; current and past, or current and a usual pattern.

For the visualizations of data over a number of days, the clinicians expressed the opinion that they found the graphs showing the number of events of the usage sensor per day or per part of day were too busy and also that the visualizations did not show a comparison with the usual pattern. The visualization for the motion sensor showing the running average (Figure 8: 7.2.1) was preferred. We note the clinicians incorrectly referred to this running average as a usual pattern. A further suggestion was to show dates on the horizontal axis, rather than the number of days since the start of monitoring.

Some of the feature names chosen by the researchers, such as *Total period in bed*, were not clear to the clinicians and caused confusion when considering the visualizations such as Figure 9: 3.1.1 for the summarization over a number of days. We sought to clarify names where possible and provide a description of each feature. A further suggestion was to include the usual pattern superimposed for the remaining two features (*Total time in bed* and *Total time out of bed*). Having the four features (*Total time in bed*, *Total time out of bed*, *Total period in bed*, and *Continuity*) together on a common visualization was deemed overly complex for daily use by the clinicians. However similar visualizations of features determined for the motion sensors were commented on as useful. The main difference between the visualizations was that the motion sensor had fewer features and was thus less busy.

### Cycle 2

We considered the feedback from the first evaluation and amended the visualizations. In general we were able to respond to all of the comments and made modifications to the visualizations to address the issues. Modifications based on the comments in cycle 1 were made as follows, and in October 2013 we held a second evaluation.

A representative set of data was selected to include a complication or intervention, so that the clinicians might have a number of examples with events in order that the effects of visualization could be considered for their clarity.

The visualization for the 24 hour usage sensor data was modified to include the usual pattern for comparison. Two alternative visualizations were developed, one had the graph of the difference from the usual pattern shown in a separate color (Figure 5: 1.3.2); the second (1.4.2; not shown) had solid vertical lines to show the upper and lower bounds of the usual pattern.

The visualization for the 24 hour motion sensor, showing the number of events per hour, was modified to include the usual pattern shown as bars of a different color adjacent to its respective actual value (Figure 7: 2.4.2).

The visualization of the number of events per part day (Figure 8: 7.2.2) was modified to include dates on the time axis in place of the number of days since entering the pilot.

The visualization for the number of days was modified to include lines for the usual pattern and standard deviation (Figure 9: 3.1.2).

**Figure 10:**
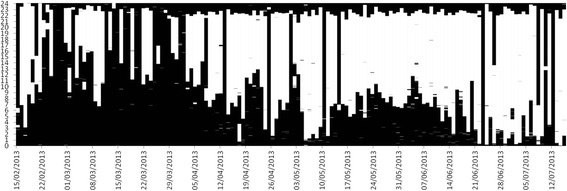
**Visualization of raw data for the bed usage sensor as stacked data (one vertical strip for each day) with ‘in use’ as black and ‘not in use’ as white (5.1.1).**

Finally, visualizations were made to display data from a sensor over a period of number of days. This was displayed with black to show activity of the sensor (such as in bed) and white to show lack of activity of the sensor (such as not in bed). Each 24 hour period was shown as a vertical strip with the 24 hour strips arranged side by side for the entire period. The date was shown on the horizontal axis, and the time of day on the vertical axis (Figure 10: 5.1.1). For the motion sensor a continuous function was created to denote presence and absence.

The second evaluation cycle was conducted as a focus group. Qualitative summary feedback was recorded and questionnaires were used to determine quantitative feedback on effectiveness. A Likert scale was used with a value between 1 (very low) and 5 (very high). The result of the quantitative feedback is shown in Table 4. We report both a non-weighted and a weighted average, in which we apply double weight for clinicians. Sections not scored were discarded from the average values.

**Table 4:** **Effectiveness scoring of visualizations showing non-weighed and weighed averages of evaluation 2**

**Identifier**	**Description**	**Non-weighed**	**Weighed**
1.3.2	24 hour usage activity colors	1.8	1.7
1.4.2	24 hour usage activity bars	2.4	2.3
2.4.2	24 hour motion events / hour	3.2	3.1
3.1.2	Days usage occupancy times	2.4	2.4
3.2.2	Days usage occupancy hours	2.8	2.6
3.3.2	Days usage occupancy only hours	3.6	3.4
3.4.2	Days usage non-occupancy hours	3.6	3.4
4.1.2	Days usage # events	3.6	3.7
5.1.2	Raw usage occupancy	3.3	3.4
6.1.2	Days motion nocturnal inactivity times	2.8	3.0
6.2.2	Days motion nocturnal inactivity hours	3.4	3.4
7.1.2	Days motion # events	3.2	3.3
8.1.2	Raw motion # events / hour	2.8	3.0
8.2.2	Raw motion activity 15 minute window	1.5	1.4
8.3.2	Raw motion activity 30 minute window	1.5	1.4

The feedback on the modified visualization of the 24 hour usage sensor data (Figure 5: 1.3.2), was that this was not intuitive and rather confusing. A suggestion was to display the actual data in one color and superimpose the usual pattern in a different color.

The feedback on the visualization of the 24 hour motion sensor data (showing the number of events per hour) and having the usual pattern as a bar adjacent to the respective actual data (Figure 7: 2.4.2) was described as not good. It was suggested that a plot of the difference between the actual data and the usual pattern might be clearer and more intuitive, as it was considered more important to see the difference than the usual pattern.

The feedback on the visualization of features for both the usage (Figure 9: 3.1.2) and motion sensors, was that they looked very busy, were not intuitive, and therefore might be difficult to use. A particular comment by the clinician was that one of the visualizations looked like an EEG (Electro Encephalogram), with a further comment, ‘once I see it as an EEG, I am stuck’. One suggestion was to replace the actual data, currently shown as a line, by a ‘trend’ line, or filtered average, and superimpose the actual data as points.

The feedback on the visualization of the activity of the usage sensor, which did include an average line, was considered good. However, the feedback on the visualization of the number of events of the motion sensor (Figure 8: 7.2.2) was that it looked ‘too busy’ and the suggestion was to plot only the actual data points, and provide the average value as a solid line. It was suggested that a stacked graph showing the total number of hours (of Time in bed and Time not in bed during the night) could be used for the visualization of the number of hours per day for the usage sensor.

The clinicians also cited that they might have to explain or use the visualizations with people not familiar with them, and therefore being simple and intuitive would assist that activity.

There was mixed feedback on the alternative visualizations for displaying raw usage sensor events (Figure 10: 5.1.1). Some considered this approach clear and simple, whereas others did not like it. However, the average feedback score was relatively high and no suggestions were made for further improvement.

### Cycle 3

We considered the feedback and suggestions from the second evaluation and amended the visualizations. Again, in general, we were able to respond to all of the comments and made modifications to the visualizations to address the issues. In November 2013 we held the third and final evaluation. Again we used a focus group and collected comments for qualitative feedback and used a questionnaire with a Likert scale for quantitative feedback on each of the visualizations.

We believe that the feedback from the first two evaluations had enabled us to develop the near final form of each of the visualizations, and we used the third cycle to concentrate on making the visualizations as clear, simple and intuitive as possible.

The feedback from the first two evaluations had suggested that the general preference was for each of the visualizations to provide the underlying trend or usual template as a solid line and superimpose the actual data as points. There was expectation that all visualizations had clear labeling and legends, preferably with date on the horizontal axis in place of days in the pilot.

The visualization of the 24 hour usage sensor data was modified to show the actual sensor data in blue and the usual pattern was superimposed in dark grey (Figure 5: 1.3.3). The simplest visualization of this data had only the actual sensor data (Figure 5: 1.2.3), which is shown in blue in Figure 5: 1.3.3.

The visualization of the motion sensor was modified to superimpose the usual pattern on the actual data (Figure 7: 2.4.3) and a second visualization had only the difference values between the actual sensor data and usual pattern (Figure 7: 2.5.3).

Each of the visualizations of summary data for each day was modified to a form that included a running average, shown as a solid line, and had the actual sensor data as points (Figure 8: 7.2.3).

The visualization of the usage sensor was modified to show the running average for each of the sum of Time in bed and Time not in bed, with the Time in bed and the Time not in bed shown as solid lines and the actual sensor data as points (Figure 9: 3.1.3). A further visualization was made showing a simplified stacked area graph of the running average values of Time in and Time out of bed (Figure 9: 3.6.3).

The third evaluation cycle was again conducted as a focus group. Qualitative summary feedback was recorded, and a questionnaire used to gain a quantitative score of effectiveness. In this evaluation, visualizations from previous iterations were also shown to the participants to allow comparison. The results of effectiveness are shown in Table 5. Discarding visualization 3.5.3, this results in the final visualizations considered most effective.

**Table 5:** **Effectiveness scoring of visualizations showing non-weighed and weighed averages of evaluation 3**

**Identifier**	**Description**	**Non-weighed**	**Weighed**
1.3.3	24 hour usage activity colors	3.7	3.8
1.2.3	24 hour usage activity simple	5.0	5.0
2.4.3	24 hour motion events / hour	4.3	4.3
2.5.3	24 hour motion events / hour differences	5.0	5.0
3.1.3	Days usage occupancy times	4.3	4.3
3.2.3	Days usage occupancy hours	5.0	5.0
3.3.3	Days usage occupancy only hours	5.0	5.0
3.4.3	Days usage non-occupancy hours	5.0	5.0
3.5.3	Days usage occupancy hours stacked	2.7	2.5
3.6.3	Days usage occupancy hours stacked areas	5.0	5.0
4.1.3	Days usage # events	5.0	5.0
6.1.3	Days motion nocturnal inactivity times	4.3	4.3
6.2.3	Days motion nocturnal inactivity hours	4.3	4.3
7.1.3	Days motion # events	4.7	4.5

In general the visualizations showing the running average values as solid lines and the actual data values as points were much preferred, and was indicated in both the comments and the scores.

However for 24 hour usage sensor data, the visualization showing only the actual sensor data without the usual pattern was preferred (Figure 5: 1.2.3). For 24 hour motion sensor data, the visualization showing only differences (Figure 7: 2.5.3) was preferred, with comments that it was easier and faster to assimilate.

For the usage sensor features, the visualization showing the separate lines (Figure 9: 3.1.3) was considered confusing, with the comment that the total line did not add information. However, the visualization of stacked areas (Figure 9: 3.6.3) was considered very good; with the comment that it was ‘easier to see’ and ‘best so far’. Note that this visualization did not contain the actual sensor data, rather, only the running average values.

For the number of hours of the usage sensor, the visualization that had each feature in a separate graph was preferred over the visualization that had all the features on a single graph, with the comment that all the separate visualizations should be visible on the screen at the same time.

A comment was made that it would be important to be able to modify the period over which the running average was calculated.

## Discussion

### Visualization development using the UCD methodology

The overall requirement of this research was to provide visualizations of habits data that were clear, simple and intuitive, and support rapid assimilation of changes from usual behavior. There were a number of further requirements for the visualizations, such as that they should show the time of any clinical event or intervention, in order to provide context and help explain events that might be evident in the sensor data.

The UCD methodology was adopted to develop visualization of sensor data for use by clinicians to assess for changes in health status. This involves undertaking several design and evaluation cycles, each intended to iterate towards an optimized solution. In our approach, we used each cycle to serve a separate purpose.

In the first design cycle we developed many different visualizations, with the intent that the most effective would be selected to be taken forward for further development. We also sought suggestions and ideas based on the visualizations to incorporate into subsequent designs.

In the second design cycle, the focus was on improving the selected visualizations and incorporating improvements based on the suggestions and feedback from the first evaluation. We produced a number of variants of each of the visualizations to explore options and elicit feedback that might be more useful in this cycle. Feedback from the second evaluation indicated that the visualizations were much improved, as evidenced from the consistent comments, specific suggestions and improved scores. We also noted that the clinicians had a clearer understanding what was expected from them and had a greater feeling for what they desired. The effectiveness scoring provided information to allow us to judge between the variants to determine the aspects that would help improve design of the visualizations.

In the third design cycle, the focus was on improving clarity and legibility of each visualization. The quantitative scoring of the third evaluation showed that significant improvement had been achieved in how effective the clinicians considered the final visualizations. Moreover, the high scores and few feedback comments indicated that no further design cycles were needed.

### Evaluation of outcomes

During our development, we noticed some important differences between the approach and attitude of the researchers and clinicians. Whereas the researchers would prefer to have as many features and as much information as possible in each visualization, the clinicians would prefer clarity and simplicity, often each visualization having only a single feature, with several visualizations per page. In addition, concepts considered intuitive to the researchers were not always to the clinicians. As the clinicians also cited that they might have to explain or use the visualizations with people not familiar with them, and therefore being simple and intuitive would assist that activity.

During the development of the visualizations, the clinicians often had issues with the meaning and interpretation of features considered important and insightful by the researchers; the clinicians considered them unclear and ambiguous, or did not understand their significance. Clear choice of name and labeling was essential.

Problems of clarity in expression and communication were also encountered; the researchers often having specific interpretation of terms, whereas clinicians using terms interchangeably. For example clinicians did not distinguish between a “usual template” (pattern) and a “moving average”.

The results revealed that the clinicians were often looking for differences between values (current and past, or current and a usual or average pattern) and therefore visualizations that accentuated this aspect were preferred. In consequence, the 24 hour visualization of motion sensor events per hour (Figure 7: 2.4.2) was often criticized as it did not show differences well. Therefore the final visualization showing the differences (Figure 7: 2.5.3) was preferred and scored highly.

There was a constant comment with highly dispersed data that was graphed as a line (such as Figure 8 (7.2.2) and Figure 9 (3.1.2)) that it was ‘too busy’, and for these cases the clinicians preferred to see filtered data, such as a running average. Where actual sensor data was to be visualized, depicting only as points and graphing the filtered data as a line provided a solution that the clinicians much preferred.

## Conclusion

The UCD method was successfully employed to converge to a design that met the main objective of this study. The resulting visualizations of relevant features that were extracted from limited sensor data, were considered highly effective and intuitive to the clinicians, and were considered suitable for them to be able to monitor the behavior patterns of patients.

The researchers recommend the approach to be applied to similar fields; employing the UCD method, with perhaps more clinicians participating in the evaluation, and involving them more closely in the design process.

We acknowledge that patient behavior would be studied more accurately by using a larger number of motion sensors around the home, but even the limited number of sensors was found to provide significant information. Bed usage sensors gave richer and more reliable data for nocturnal activity.

## Additional file

Additional file 1:
**Individual Scoring sheet.** Data visualisation scoring.Scoring: Effectiveness of visualisation (1 = very low … 5 = very high).
