# Supplementary material for: Designing effective visualizations of habits data to aid clinical decision making
Source: BMC Med Inform Decis Mak. 2014 Nov 30;14:102. doi: 10.1186/s12911-014-0102-x (PMC4265320; doi:10.1186/s12911-014-0102-x)
Supplement: Additional file 1: — Individual Scoring sheet. Data visualisation scoring.Scoring: Effectiveness of visualisation (1 = very low … 5 = very high). [file 12911_2014_102_MOESM1_ESM.doc]

# Additional file 1. Individual Scoring sheet

**Data visualisation scoring**

Scoring: Effectiveness of visualisation (1 = very low … 5 = very high)

| **Slide number** | **1** | **2** | **3** | **4** | **5** | **Comments** |
| --- | --- | --- | --- | --- | --- | --- |
|  |  |  |  |  |  |  |
|  |  |  |  |  |  |  |
|  |  |  |  |  |  |  |
